# Combined Effects of Cellulose Nanofiber Nucleation and Maleated Polylactic Acid Compatibilization on the Crystallization Kinetic and Mechanical Properties of Polylactic Acid Nanocomposite

**DOI:** 10.3390/polym13193226

**Published:** 2021-09-23

**Authors:** Siti Shazra Shazleen, Lawrence Yee Foong Ng, Nor Azowa Ibrahim, Mohd Ali Hassan, Hidayah Ariffin

**Affiliations:** 1 Laboratory of Biopolymer and Derivatives, Institute of Tropical Forestry and Forest Products (INTROP), Universiti Putra Malaysia, Serdang 43400, Malaysia; shazra.shazleen@yahoo.com (S.S.S.); lawrenceyfng@gmail.com (L.Y.F.N.); 2 Department of Chemistry, Faculty of Science, Universiti Putra Malaysia, Serdang 43400, Malaysia; norazowa@upm.edu.my; 3 Department of Bioprocess Technology, Faculty of Biotechnology and Biomolecular Sciences, Universiti Putra Malaysia, Serdang 43400, Malaysia; alihas@upm.edu.my

**Keywords:** cellulose nanofiber, nucleating agent, crystallization rate, polylactic acid, compatibilizer

## Abstract

This work investigated the combined effects of CNF nucleation (3 wt.%) and PLA-*g*-MA compatibilization at different loadings (1–4 wt.%) on the crystallization kinetics and mechanical properties of polylactic acid (PLA). A crystallization kinetics study was done through isothermal and non-isothermal crystallization kinetics using differential scanning calorimetry (DSC) analysis. It was shown that PLA-*g*-MA had some effect on nucleation as exhibited by the value of crystallization half time and crystallization rate of the PLA/PLA-*g*-MA, which were increased by 180% and 172%, respectively, as compared to neat PLA when isothermally melt crystallized at 100 °C. Nevertheless, the presence of PLA-*g*-MA in PLA/PLA-*g*-MA/CNF3 nanocomposites did not improve the crystallization rate compared to that of uncompatibilized PLA/CNF3. Tensile strength was reduced with the increased amount of PLA-*g*-MA. Contrarily, Young’s modulus values showed drastic increment compared to the neat PLA, showing that the addition of the PLA-*g*-MA contributed to the rigidity of the PLA nanocomposites. Overall, it can be concluded that PLA/CNF nanocomposite has good performance, whereby the addition of PLA-*g*-MA in PLA/CNF may not be necessary for improving both the crystallization kinetics and tensile strength. The addition of PLA-*g*-MA may be needed to produce rigid nanocomposites; nevertheless, in this case, the crystallization rate of the material needs to be compromised.

## 1. Introduction

Most items in the market are packed for various purposes such as protection, storage, preservation, transportation and presentation. The consumption of packaging is high in both food and non-food industries. A wide range of materials can be used for packaging applications such as glass, paper and petroleum-based polymers. In regard to their versatility and outstanding characteristics, petroleum-based plastics have been widely used for various purposes. Nevertheless, these plastics can only be used once before they are thrown out, as these materials are non-biodegradable and recycling or reusing them is a very challenging task, causing the generation of a huge amount of non-biodegradable wastes [1]. Therefore, the use of these polymers has been restricted due to the expensive recycling methods and limitations of disposal methods as well as adequate landfill problems [2]. By shifting to biodegradable polymers to replace synthetic polymers, environmental pollution caused by the accumulation of non-biodegradable synthetic plastics can be reduced significantly. Moreover, consumer demand for products that are environmentally friendly, safer and non-toxic along with a currently advantageous economic scenario leads to the assumption that biodegradable packaging materials will become increasingly prevalent [3]. Hence, biodegradable polymers are often used as an alternate material to replace synthetic polymers.

Biodegradable polymers have attracted many attentions as a potential replacement for petro-plastic packaging, but it is relatively poor material since most of it is made from biological sources and their properties are not as excellent as synthetic plastic, which limits its usage in industry [4]. Nevertheless, polylactic acid (PLA) polymer has gained a lot of interest as one of the most promising alternatives to synthetic polymers, as its properties are comparable to those of polystyrene and, as a result, it is used in large quantities, particularly in packaging applications [5,6,7].

PLA is a versatile material and has been favored because it can be mass-produced from renewable agricultural raw materials that are fermented into lactic acid, thereby reducing the reliance of society on petrochemical feedstocks. PLA can be considered as an attractive sustainable alternative to petroleum-based plastic since it can be processed using the same machinery where it can be molded, vacuum formed, blown or extruded as easily as conventional plastic [8]. Nevertheless, the use of PLA in commercial applications is still limited mainly due to its high production cost, brittleness, low thermal stability and low gas barrier properties [9]. Additionally, PLA has a slow crystallization rate due to high molecular weight that makes it relatively difficult to utilize as a main material using a common industrial process such as injection molding due to molded PLA mostly amorphous in these rapid processes. Therefore, PLA processing requires longer molding cycles and causes difficulties in demolding section [10]. It is crucial to enhance the crystallization rate of PLA and optimize it to achieve the desired final properties of products for industrial applications. These limitations give a negative effect on the crystallization and mechanical properties of the PLA. Many have attempted to overcome these limitations by various methods, the most common being the addition of reinforcing materials which also can act as nucleating agents such as graphene [11,12], talc [13,14,15] and natural fibers [16,17,18,19]. These additives have been successfully used as nucleating agents and have proven to show an enhancement in the crystallization kinetics and mechanical properties of PLA.

Nanocellulose has recently gained much attention due to its outstanding properties and applicability in various fields. In fact, nanocellulose is expected to have great potential to replace many non-renewable materials as cellulose materials are the most abundant biological and multifunctional raw materials that can also be self-assembled into well-defined multi-scale architectures, from micro to nanosize [20]. One of the most widely utilized nanocellulose is cellulose nanofiber (CNF) including as reinforcement in nanocomposites [21,22,23,24,25,26,27,28] and can also act as a nucleating agent for improving crystallization behaviors of the polymer matrix [6,29,30,31]. CNF has been proven to be an effective nucleating agent to enhance the crystallization behaviors of the polymer matrix as compared to microsized cellulose fibers [30]. CNF allows for a heterogeneous nucleation process that promotes a decrease of the free energy barrier and accelerates the crystallization rate [9]. The addition of CNF could also increase the number of nucleation sites and decrease the crystal size with an increased nucleation density [32]. Moreover, CNF has been utilized globally in industrial application owing to its outstanding properties including; high flexibility, high in mechanical strength, high crystallinity, high aspect ratio and renewability that can be produced by mechanical or chemical treatments that are advantageous for nanocomposites and reinforced polymers [33,34].

Although CNF has gained substantial market interest as an alternative to inorganic nano-sized reinforcements in nanocomposite materials, the main challenge of using nanofiber is the difficulties of achieving good nanofiber dispersion in a non-polar medium due to their polar surfaces [35]. CNF has been shown to be compatible with PLA based on previous studies, but agglomeration was observed to occur at a certain amount of CNF, notably at higher loading. Difficulties of PLA to interact with reinforcements containing polar moieties owing to its non-polar nature resulting in poor nanofiller dispersion, insufficient adhesion and reduced its properties. This creates a major drawback for PLA nanocomposites because they are unable to achieve their maximum performance ability. To extent their industrial applications, CNF must overcome their weak interfacial compatibility with PLA matrix. An alternative approach is to blend the PLA matrix with CNF in the presence of compatibilizer. Compatibilization method has the capability to improve the mechanical properties by grafting monomers to the polymer backbone [36]. Compatibilizer can be produced through free-radical copolymerization or grafting of the reactive group onto polymer chains during the compounding process [37]. Several compatibilizers have been studied and used in previous researches to compatible with PLA blends (i.e., starch, talc) including dioctyl maleate (DOM) [38], methylenediphenyl diisocyanate (MDI) [32,39,40,41] and glycidyl methacrylate (GMA) grafted PLA (PLA-*g*-GMA) [42].

Nowadays, maleated PLA (PLA-*g*-MA) has been widely used as a compatibilizer between PLA and CNF. PLA-*g*-MA can be prepared by grafting maleic anyhydride (MA) onto PLA chains in the presence of peroxides as an initiator to create reactive sites for MA to graft. Compatibilization achieved by the addition of PLA-*g*-MA produced through reactive extrusion is an effective way of manufacturing nanocomposites at high production rates and therefore economically feasible on an industrial scale [43]. Previous research conducted by Ghasemi et al. [44] has observed improvements in tensile strength, modulus and impact strength of PLA/CNF5 by 55, 15 and 40 while PLA/CNF5/PLA-*g*-MA5 by 169, 40 and 131% as compared to neat PLA, respectively. This increment might be attributed to compatibilizer impact on enhancing interfacial adhesion between two phases: CNF and PLA matrix. Additionally, the results obtained from heat distortion temperature study indicated that the addition of PLA-*g*-MA had a positive effect on the thermal stability of PLA nanocomposites.

In the previous research, we investigated the optimal PLA and CNF blending ratio to achieve the maximal crystallization rate and mechanical properties for PLA nanocomposites [45]. In this article, we study the PLA nanocomposites blended with 3 wt.% of CNF with the addition of PLA-*g*-MA as a compatibilizer. It is evident from the literature review that no research has been conducted on the combination effects of nucleation and compatibilization between CNF and PLA-*g*-MA and their correlation to improve the crystallization and mechanical properties of PLA. Hence, in this study we intend to enhance the crystallization and compatibility of nanocomposites through the combination effects of CNF nucleation and PLA-*g*-MA compatibilization to increase the crystallization rate and to optimize the interfacial interaction between PLA and CNF to improve the mechanical performances.

## 2. Materials and Methods

### 2.1. Materials

Polylactic acid (PLA) (grade 2003D, NatureWorks LLC, Minnetonka, MN, USA) in pellet form was purchased from Ecoscience Sdn. Bhd with melt flow index of 6.0 g/10 min at 210 °C was used as is. The number- and weight-average molecular weights (M_n_ and M_w, respectively_) and polydispersity index (PDI; calculated as the ratio of M_w_/M_n_) of 165,189 g/mol, 76,066 g/mol and 2.17 was determined by gel permeation chromatograpy (GPC) at 40 °C using THF as eluant on a Waters apparatus (USA) equipped with three columns Styragel HR1, HR3 and HR5 and with a Waters 2414 refractive index detector at an eluation rate of 1 mL/min. The system was calibrated using polystyrene standards. Cellulose nanofiber (CNF) with a concentration of 2 wt.% fiber slurry was purchased from ZoepNano Sdn. Bhd. Maleic anhydride (MA) and dibenzoyl peroxide (DBPO) were purchased from Synergy Scientific Sdn. Bhd, while methanol, chloroform and hydrochloric acid (HCl) were purchased from Greenfinite Sdn. Bhd. All chemicals were used as received.

### 2.2. Methods

MA was grafted onto PLA by the melt-blending method using an internal mixer, Brabender Plasticoder (Brabender Messtechnik GmbH Co., Duisburg, Germany) at 160 °C for 15 min with a rotor speed of 70 rpm, in accordance with the method modified from Birnin-yauri et al. [46] and Calderón et al. [37]. PLA resin was oven-dried at 60 °C overnight prior to mixing to prevent hydrolytic degradation melt compounding. The compatibilizer at 3 and 10 wt.% of MA were prepared by melting the oven-dried PLA resins for 5 min followed by the addition of DBPO as an initiator and mixed for an additional 2 min. Finally, MA was added and mixed continuously for another 8 min, resulting in the production of PLA-g-MA. The composition of PLA, MA and DBPO are tabulated in Table 1.

The PLA-*g*-MA, yellow color strips was removed and then ground into small pieces with a grinder. The unreacted MA and DBPO were eliminated from the compatibilizer by placing it in a vacuum oven at 80 °C overnight.

Purification was done by dissolving 2.0 g of PLA-*g*-MA in 40 mL chloroform, followed by addition of 0.75 mL of 1 M HCl at room temperature to hydrolyze the anhydride into carboxylic acid. The mixture was then stirred for 1 h and drop-wise added into 400 mL methanol. The filtered precipitate was rinsed with methanol several times, and vacuum oven-dried at 80 °C overnight. Next, direct titration was carried out to determine the degree of MA grafting onto PLA. A total of 0.4 g of the purified sample was dissolved in 20 mL chloroform and titrated against 0.04 M methanolic NaOH using phenolphthalein as an indicator. Degree of grafting was calculated using the equation:

(1) $$\mathrm{MA}\mathrm{grafting}\;(\%)=\frac{N_{\mathrm{NaOH}}\cdot V_{\mathrm{NaOH}}}{2\times m_{\mathrm{sample}}}\times 98.06\times 100$$

where N_NaOH_ and V_NaOH_ indicate the volume (L) and normality (mol/L) of NaOH and m_sample_ is the sample weight (g). The sample was determined three times and the average degree of grafting was reported.

The grafting efficiency of PLA-*g*-MA was calculated using the equation:

(2) $$\mathrm{Grafting}\;\mathrm{efficiency}\;(\%)=\frac{\mathrm{weight}\;\mathrm{of}\;\mathrm{grafted}\;\mathrm{monomer}}{\mathrm{weight}\;\mathrm{of}\;\mathrm{loaded}\;\mathrm{monomer}}\times 100$$

where the weight of the grafted monomer was obtained by multiplying MA grafting percentage with sample weight (m_sample_) and the weight of loaded monomer indicate the feeding weight of anhydride monomers.

Fourier transform infrared spectroscopy (FTIR) was conducted on the PLA pellet, pure MA and purified PLA-*g*-MA to monitor the grafting process of MA onto PLA backbone using a Perkin Elmer Spectrum 100 series spectrophotometer (Waltham, MA, USA), which was equipped with an attenuated total reflectance (ATR) capacity at a wavenumber range of 500–4000 cm^−1^.

Solid-state ^13^C NMR nuclear magnetic resonance spectra were recorded on a 500 MHz NMR spectrometer (JOEL, model JNM-ECX500). The spectra for neat PLA and purified PLA-*g*-MA (10% of MA) were obtained by 2048 scans in a spectral width of 200 ppm and the number of raw data points was 6144.

#### Preparation of Nanocomposites

Nanocomposites neat PLA, PLA/PLA-*g*-MA3/CNF0 and PLA/PLA-*g*-MA/CNF3 with different PLA-*g*-MA loading were prepared using the same method as described in our previous study Shazleen et al. [45]. Prior to mixing, moisture from PLA pellets was removed by drying at 60 °C for 24 h under vacuum condition to minimize hydrolytic degradation during the melt compounding at high temperature. The dried PLA pellets were mixed with 3 wt.% CNF and PLA-*g*-MA at different contents (1–4 wt.%) at 170 °C for 30 min with a rotor speed of 70 rpm. The composition of PLA, PLA-*g*-MA and CNF for nanocomposites are as tabulated in Table 2.

### 2.3. Characterization

#### 2.3.1. Crystallization Kinetics Analysis

Non-isothermal and isothermal crystallization behaviors of the nanocomposites were characterized using DSC (Q200, TA Instruments, New Castle, DE, USA). Indium was used as standard calibration for temperature and heat of fusion. Sample weighing between 7 to 10 mg was used for the measurement.

For non-isothermal crystallization, the samples were first heated from 30 °C to 190 °C at a rate of 2 °C/min and maintained at this temperature for 3 min to remove the prior thermal history of the samples. The samples were then cooled to −40 °C at the same rate and held at that temperature for 3 min to evaluate their ability to crystallize upon cooling. Subsequently, the samples were reheated to 190 °C at the same rate.

For isothermal crystallization, the samples were firstly heated to 190 °C at a rate of 10 °C/min and maintained at this temperature for 3 min to remove their prior thermal history. The samples were then rapidly cooled to the isothermal crystallization temperature of 90, 100 and 110 °C at a rate of 50 °C/min and maintained at this temperature until the crystallization process in the samples was completed.

#### 2.3.2. Mechanical Analysis

Tensile strength (MPa), elongation at break (%), and Young’s modulus (GPa) were measured by using an Instron 5566 Universal Testing Machine (Norwood, MA, USA) with a load cell of 10 kN and crosshead speed of 5 mm/min at room temperature. Five dog-bone shaped specimens, each with a 3 mm thickness, were tested according to the standard method of ASTM D 638-05.

#### 2.3.3. Morphological Analysis

The morphology of the fracture surfaces of the neat PLA, PLA/PLA-*g*-MA3/CNF0 and PLA/PLA-*g*-MA/CNF3 nanocomposites from tensile testing were observed using a field emission scanning electron microscope FESEM (FEI Nova NanoSEM 230, Hillsborough, OR, USA). The acceleration voltage used was 5 kV, and samples were sputter-coated with gold prior to FESEM observation to avoid charging.

## 3. Results and Discussion

### 3.1. Grafting Analysis and Characterization of PLA-g-MA

The grafting percentage and efficiency of PLA-*g*-MA samples with 3 and 10 wt.% of MA content were determined by the titration method and the data obtained were tabulated in Table 3.

Findings from this analysis revealed that the grafting percentage and efficiency increased by increasing the MA content. The addition of 3 wt.% MA in PLA resulted in 0.15% of MA grafting and 3.13% grafting efficiency. The reason behind the low percentage of MA grafting was due to insufficient amount of MA that could be grafted onto PLA chain, thus leading to low grafting efficiency. Further increasing the MA content to 10 wt.% resulted in increasing the grafting degree and efficiency to 2.10 and 13.2%, respectively. This shows that increasing the MA content able to increase the possibility of PLA macroradicals generated from the reaction between PLA and DBPO (initiator) to interact with MA to form MA grafted PLA and thus increasing the grafting degree and efficiency [47]. This observation was also reported by Syazana et al. [48]. Muenprasat et al. [49] found that the grafting percentage was not significantly changed when the MA concentration exceeded 10 wt.% that might be attributed with the occurrence of PLA chain scission reaction at higher MA content and it could compete with the grafting reaction. Since the composition of the DBPO was constant in this study, any variations in grafting degree is attributable to the changes in MA content. The PLA-g-MA with the highest grafting degree and efficiency was used to prepare PLA/PLA-g-MA/CNF3 nanocomposites.

**Figure 1:**
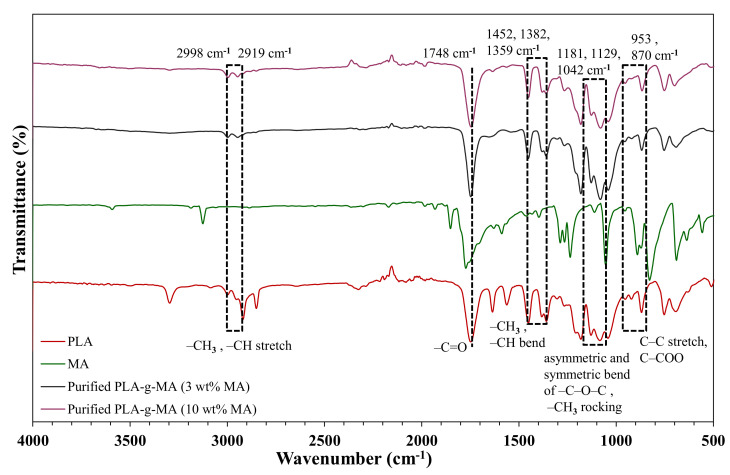
FTIR spectra of neat PLA, pure MA and purified PLA-*g*-MA at 3 and 10 wt.% of MA.

The grafting reaction of MA on PLA backbone has been observed through FTIR analysis where it was performed on neat PLA, pure MA and purified PLA-*g*-MA samples. The FTIR spectra are depicted in Figure 1.

Neat PLA exhibit characteristic transitions at 2998 and 2919 cm^−1^ (-CH_3_ and -CH stretching vibrations), 1748 cm^−1^ (-C=O), 1452, 1382 and 1359 cm^−1^ (-CH_3_ and -CH bending vibrations), 1266 cm^−1^ (stretching vibration of -C-O-C), 1181, 1129 and 1042 cm^−1^ (asymmetric and symmetric bending vibrations of -C-O-C and -CH_3_ rocking), 953 cm^−1^ (C-C stretching vibration) and 870 cm^−1^ (C-COO) [50]. The FTIR spectra of purified PLA-*g*-MA was almost identical to FTIR spectra of neat PLA, but the only difference that can be noted was the decrease in the intensity of the absorption bands at 2998, 2947, 1452, 1382, and 1359 cm^−1^. This phenomenon could be attributed to the -CH group of PLA main-chain involved in the grafting reaction, thus confirming that the grafting reaction has occurred. Nevertheless, the absorption peaks of MA at 1780 and 1850 cm^−1^ on PLA-*g*-MA that correspond to symmetric and asymmetric stretching carbonyl groups of cyclic MA were not detected by FTIR as opposed to the spectrum of pure MA and PLA-*g*-MA that could be due to the overlapping of carbonyl stretching signal present in both grafted MA and PLA backbone [18].

**Figure 2:**
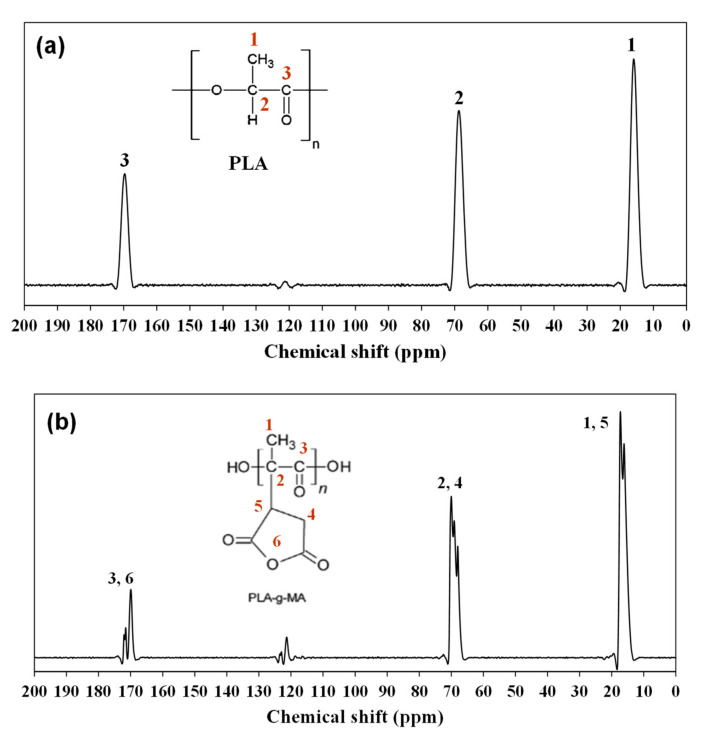
^13^C-NMR spectrum of (**a**) PLA and (**b**) purified PLA-*g*-MA at 10 wt.% of MA.

For this reason, solid state ^13^C NMR analysis has been performed to verify the occurrence of grafting reaction. PLA-*g*-MA with 10 wt.% of MA was selected due to its higher percentage grafting and efficiency. Figure 2 shows the ^13^C NMR spectrum of neat PLA and PLA-*g*-MA (10 wt.% of MA) samples.

Here, characteristic signals of PLA are observed at 169.8, 68.6 and 15.8 ppm referring to carbonyl (-C=O), methine (-CH) and methyl carbons (-CH_3_), respectively. In the PLA-*g*-MA spectrum, succinic anhydride signals (methine, -CH and methylene carbons, -CH_2_) are not observable that may be due to overlapping of their signals with PLA chemical shifts. However, the presence of MA in PLA could be indirectly demonstrated by the two peaks observed at 16.2 and 17.4 ppm as well as three peaks in the range of 68–72 ppm and 170–180 ppm. The separation of signals can be attributed to the different chemical environments of PLA resulting from the inequivalent level of grafting [51]. Gross et al. [52] report signals similar as reported in this study.

### 3.2. Non-Isothermal Crystallization Kinetics

**Figure 3:**
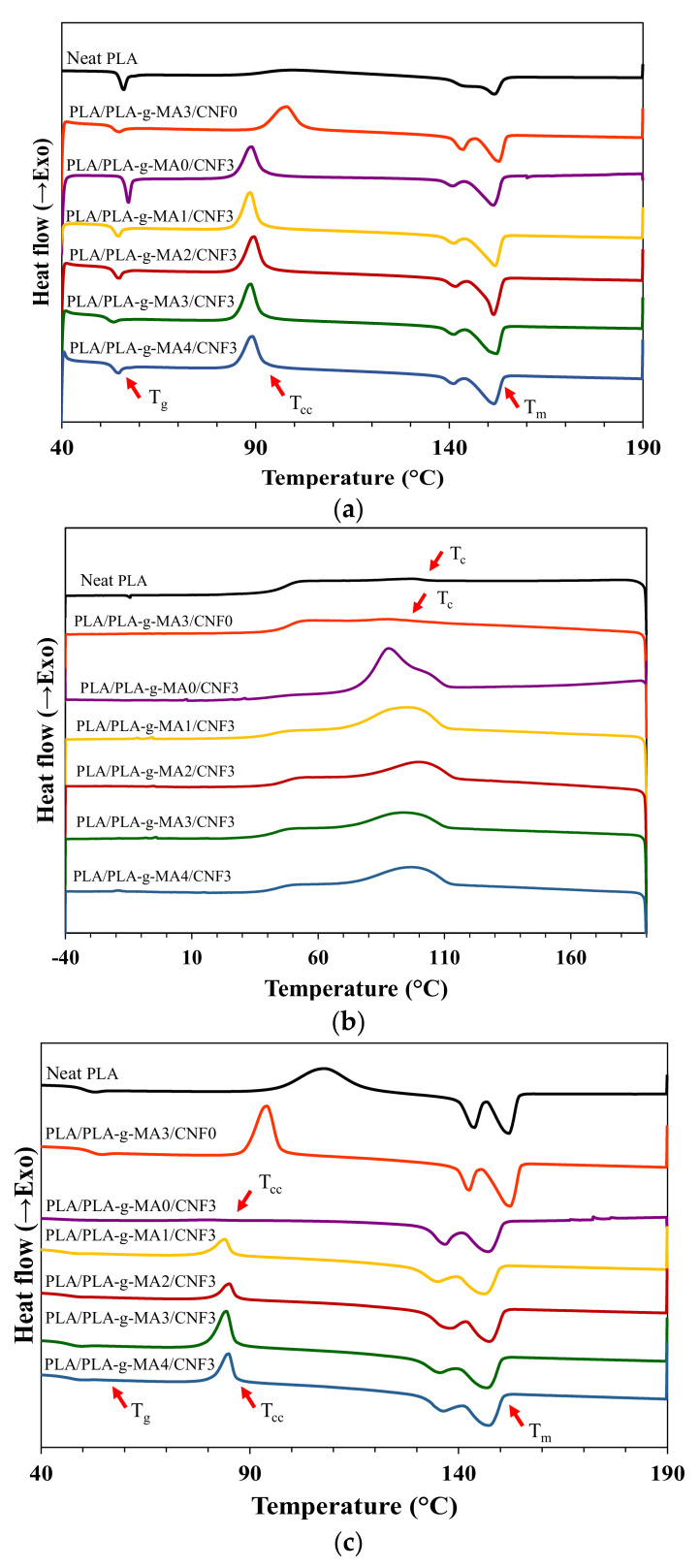
DSC curves at 2 °C/min for neat PLA, PLA/PLA-*g*-MA3/CNF0 and PLA/PLA-*g*-MA/CNF3 nanocomposites: (**a**) first heating scans, (**b**) cooling scans and (**c**) subsequent heating scan.

The combination effects of CNF nucleation and PLA-*g*-MA compatibilization at different PLA-*g*-MA content on the crystallization kinetics of PLA/CNF3 nanocomposites was compared using DSC measurements. Figure 3b,c show the non-isothermal DSC cooling curves and subsequent heating curves of neat PLA, PLA/PLA-*g*-MA3/CNF0 and PLA/PLA-*g*-MA/CNF3 nanocomposite samples at different PLA-*g*-MA content (0–4 wt.%). Table 4 summarizes thermal properties of the nanocomposites estimated from the DSC curves including glass transition temperature (T_g_), crystallization peak temperature (T_c_), cold crystallization peak temperature (T_cc_), melting peak temperatures (T_m1_, T_m2_), enthalpy of crystallization (∆H_c_), enthalpy of cold crystallization (∆H_cc_), enthalpy of fusion (∆H_m_) and degree of crystallinity (X_c_). The degree of crystallinity (X_c_) for neat PLA, PLA/PLA-g-MA3/CNF0 and PLA/PLA-g-MA/CNF3 nanocomposites were calculated as follows:

(3) $$X_{c}=\frac{\Delta H_{m}-\Delta H_{\mathrm{cc}}}{\Delta H_{m}^{{}^{\circ}}}\times 100\%$$

where ∆H_m_ is the enthalpy of melting, ∆H_cc_ is the crystallization enthalpy during the DSC scan and ∆H°_m_ is the enthalpy of melting of 100% crystalline PLA (∆H°_m_ of PLA = 93.7 J g^−1^).

It can be seen from cooling curves in Figure 3b and the data in Table 4 that the crystallization peak of neat PLA and PLA/PLA-g-MA3/CNF0 were almost unnoticeable and exhibits low crystallization enthalpy below 2 J/g. The onset crystallization temperature (T_c_) for neat PLA decreased by about 10 °C from 106.1 °C to 96.5 °C when only PLA-*g*-MA was added to the matrix indicating the introduction of PLA-*g*-MA can hinder the crystallization of PLA, which could be due to the disturbance of the regularity of PLA chains [11]. This is in contrary to the CNF addition in which it is seen that the addition of 3 wt.% CNF without the presence of PLA-g-MA led to the formation of clear and sharp crystallization peak upon cooling, with high ∆H_c_ at 33.6 J/g. The onset T_c_ was also noticeably increased by almost 8 °C, from 106.1 °C (neat PLA) to 114.6 °C. This indicates that the nucleation effect of CNF effectively accelerates crystallization process to occur during cooling as reported by Kotsilkova et al. [53]. Effect of PLA-*g*-MA addition on PLA/CNF3 nanocomposites was determined by incorporating 1–4 wt.% of PLA-g-MA in the nanocomposite. Based on Figure 3b and Table 4, it is seen that the addition of PLA-*g*-MA generally did not improve the T_c_ as compared to the uncompatibilized PLA/CNF3.

In the subsequent heating process, T_g_ of neat PLA was observed at 50.4 °C as shown in Figure 3c and Table 4. Reinforcement of 3 wt.% CNF reduced the T_g_ to 43.9 °C, indicating an increase in PLA chain mobility that resulted in better flexibility. Meanwhile, it was discovered that by solely introducing PLA-*g*-MA to PLA matrix, the T_g_ increased slightly to 51.9 °C. In the case of PLA/PLA-*g*-MA/CNF3 nanocomposites, it was found that the T_g_ increased with PLA-*g*-MA content, and the overall T_g_ for these nanocomposites was higher as compared to PLA/CNF3. This could be explained by the restriction of molecular motion when anhydride carboxyl group was grafted onto the PLA, which increased the nanocomposites rigidity. Similar observation was reported by Wu [17].

In all nanocomposites, the T_cc_ and ∆H_cc_ were lower as compared to neat PLA. This demonstrates that neat PLA had a slower crystallization process and was unable to crystallize properly during cooling (as shown in Figure 3b). Addition of CNF and PLA-*g*-MA generally reduced the ∆H_cc_, and the effect was more remarkable with the addition of CNF as compared to PLA-*g*-MA.

For melting behavior, neat PLA, PLA/PLA-g-MA3/CNF0, uncompatibilized and compatibilized PLA/CNF3 nanocomposites showed bimodal melting peaks as seen in Figure 3c with T_m1_ at the first melting point (at a lower melting temperature) and T_m2_ at the subsequent peak (at a higher melting temperature). The double endothermic peaks were attributed to melting–recrystallization–melting processes of PLA lamellae. The first peak was observed at around 135–143 °C and the other at 146–152 °C. The addition of only CNF or in combination with PLA-*g*-MA did not change melting temperatures much differently between these nanocomposites.

The degree of crystallinity, X_c_, for neat PLA, PLA/PLA-*g*-MA3/CNF0 and PLA/PLA-*g*-MA/CNF3 were calculated and tabulated in Table 4. The X_c_ of neat PLA was only 2.3%, whereas the addition of PLA-*g*-MA alone in the matrix increased nanocomposites crystallinity to 6.7%. The PLA/CNF3 alone recorded the highest X_c_ of 44.2% with an almost 95% increment relative to neat PLA. Nevertheless, the introduction of PLA-*g*-MA to PLA/CNF3 nanocomposites resulted in the reduction of crystallinity as compared to uncompatibilized sample. This might be due to irregular MA chain branching to PLA backbone, thus reduce the regularity of PLA chains and hinder the crystalline growth of PLA [54].

### 3.3. Isothermal Crystallization Kinetics

**Figure 4:**
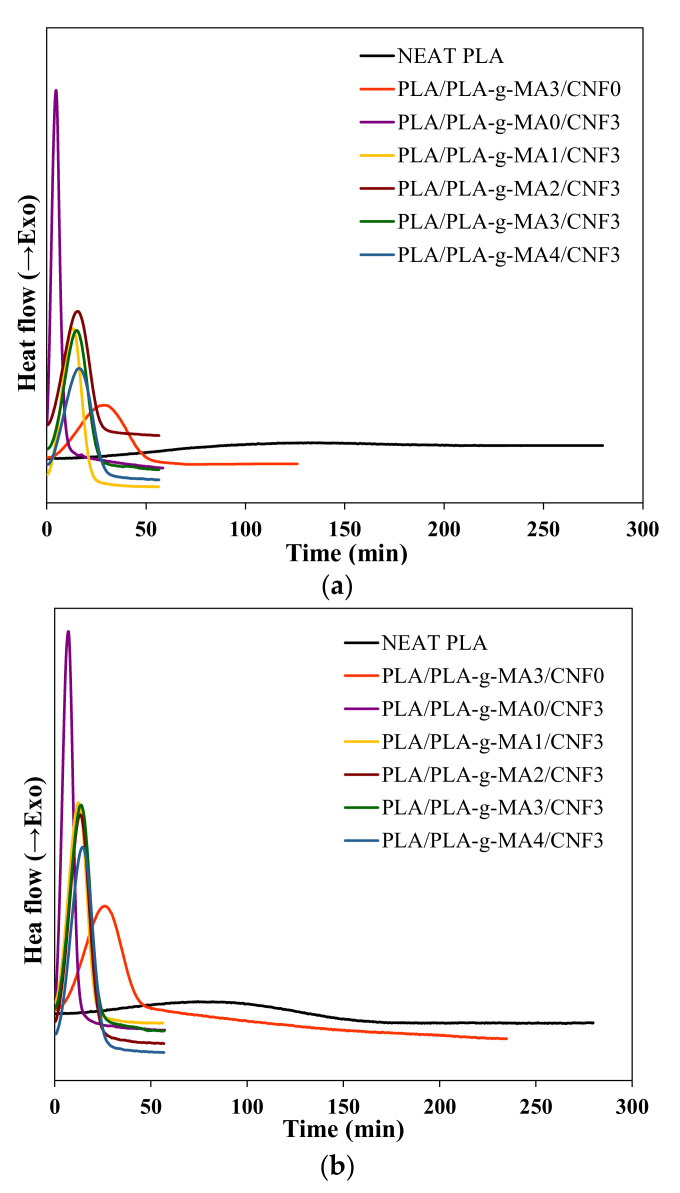

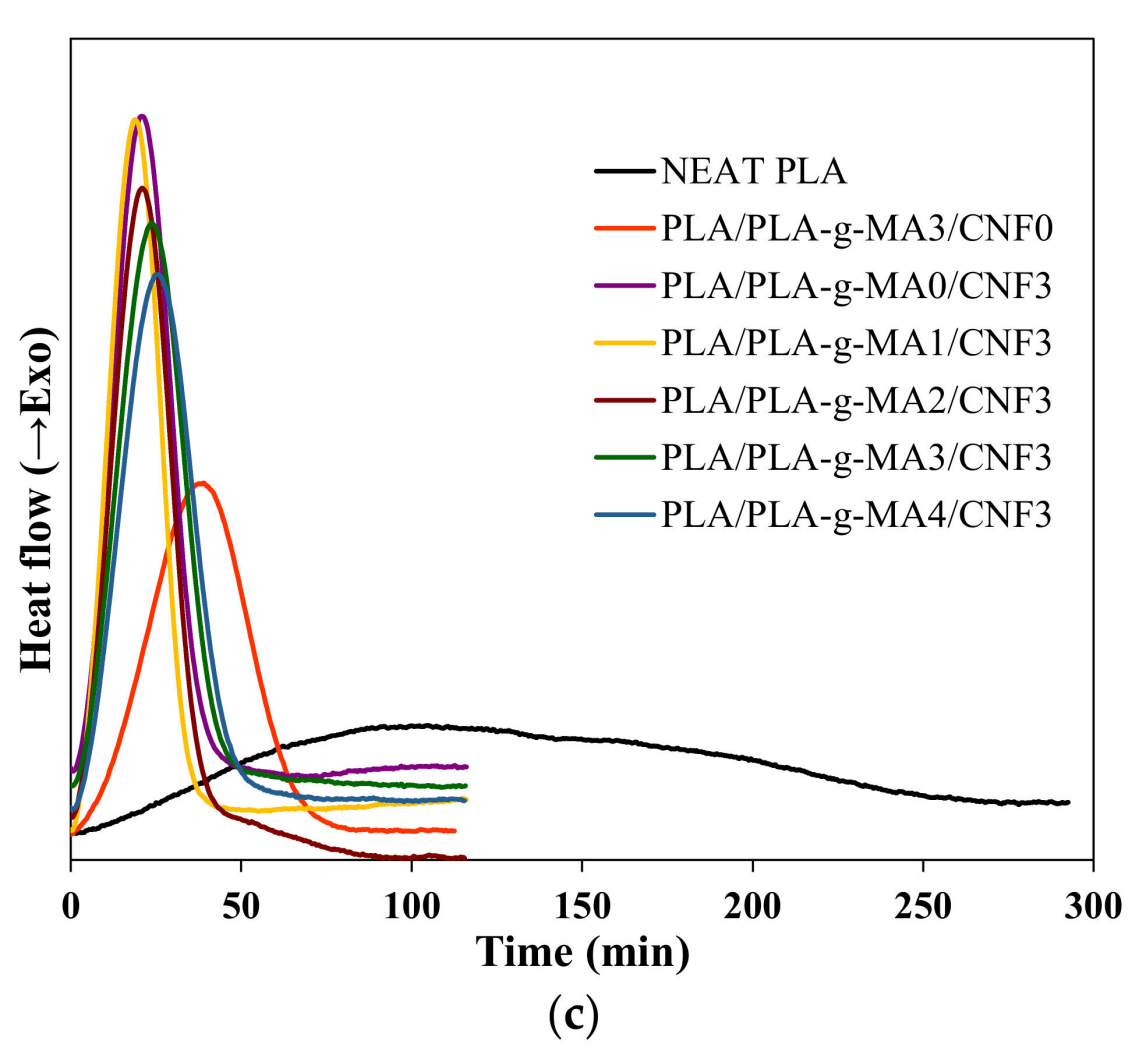
Isothermal crystallization isotherms of neat PLA, PLA/PLA-*g*-MA3/CNF0 and PLA/PLA-*g*-MA/CNF3 nanocomposites at (**a**) 90, (**b**) 100 and (**c**) 110 °C.

Isothermal crystallization analysis was performed to study the effect of CNF nucleation and PLA-*g*-MA compatibilization at different PLA-*g*-MA content on the crystallization rate of PLA reinforced with 3 wt.% of CNF. DSC curves of isothermal crystallization for neat PLA, PLA/PLA-*g*-MA3/CNF0 and PLA/PLA-*g*-MA/CNF3 nanocomposite samples were presented in Figure 4. At T_c_ = 90, 100 and 110 °C, it is obvious that the crystallization rate of neat PLA is extremely slow where there was no crystallization within the first 100 min. The introduction of PLA-*g*-MA in the PLA matrix resulted in the appearance of a sharp exothermal peak at an earlier time (60–75 min). Nevertheless, the crystallization took a longer time as compared to PLA/CNF3 (10–30 min) at T_c_ = 90, 100 and 110 °C. This may suggest that the addition of PLA-*g*-MA induces the crystallization slightly. In the case of PLA/PLA-*g*-MA/CNF3, it was observed that the time taken for complete crystallization was longer with increased amount of PLA-*g*-MA.

To analyze the isothermal crystallization kinetics, the isothermal DSC curves were integrated between t = 0 and t and divided by the overall crystallization rate to calculate the relative degree of crystallinity as follows:

(4) $$X_{\mathrm{rel}}=\frac{\int_{0}^{t}\frac{\mathrm{dH}\left(t\right)}{\mathrm{dt}}\mathrm{dt}}{\int_{0}^{\infty }\frac{\mathrm{dH}\left(t\right)}{\mathrm{dt}}\mathrm{dt}}$$

**Figure 5:**
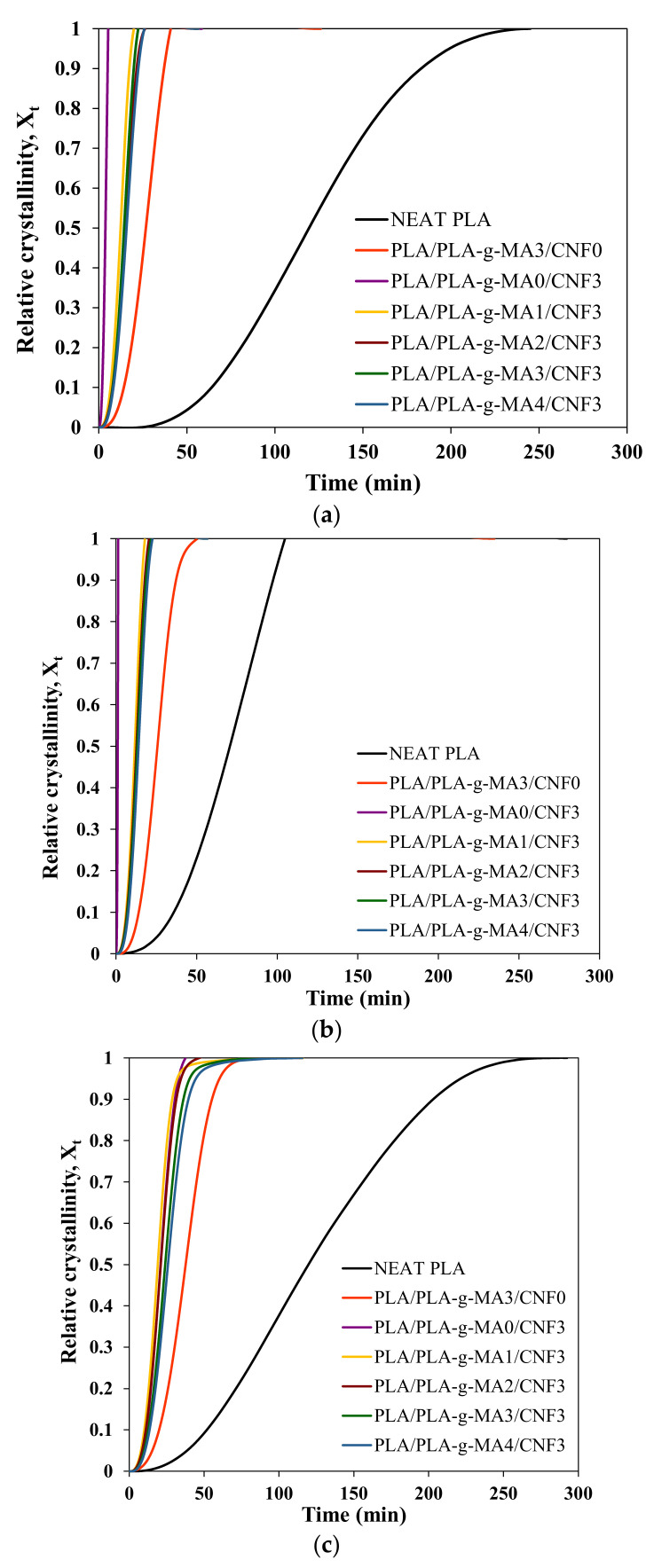
The relative crystallinity of neat PLA, PLA/PLA-*g*-MA3/CNF0 and PLA/PLA-*g*-MA/CNF3 nanocomposites at (**a**) 90, (**b**) 100 and (**c**) 110 °C.

**Figure 6:**
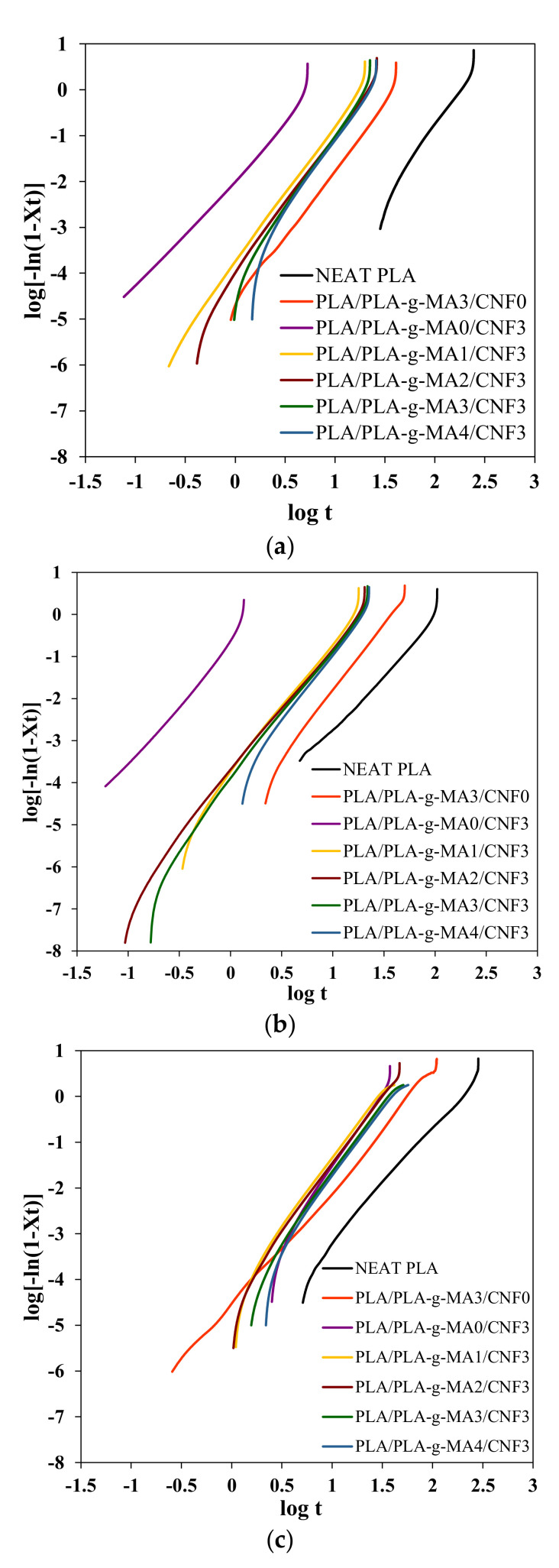
Avrami plots of neat PLA, PLA/PLA-*g*-MA3/CNF0 and PLA/PLA-*g*-MA/CNF3 nanocomposites isothermally crystallized at (**a**) 90, (**b**) 100 and (**c**) 110 °C.

Avrami equation was used to study the isothermal melt crystallization kinetics where the relative degree of crystallinity (X_rel_) was described as follows:

X_rel_ (t) = 1 − exp (−kt^n^) (5)

where n is the Avrami exponent that depends on the nature of the nucleation mechanism and growth geometry of the crystal, k is the crystallization rate constant that involves both nucleation and growth rate parameters and t is time. Equation (5) can be transformed into the double-logarithmic form,

log [−ln (1 − X_rel_(t))] = log k + n log t (6)

where the parameters n (slope) and k (y-intercept) were determined by plotting log [−ln (1 − X_rel_(t))] against log t. Figure 5 and Figure 6 show the X_t_ versus t and Avrami plots for neat PLA, PLA/PLA-*g*-MA3/CNF0 and PLA/PLA-*g*-MA/CNF3 nanocomposites containing different PLA-g-MA content isothermally melt-crystallized at 90, 100 and 110 °C. The crystallization half time t_1/2_ is another important crystallization kinetics parameter, which is defined as the time required to achieve 50% of the final crystallinity of the samples was calculated by the following equation:

(7) t1/2=ln 2k1n

Generally, the crystallization rates are expressed through the use of crystallization half time. It can be calculated by the reciprocal of t_1/2_.

(8) $$\mathrm{Crystallization}\;\mathrm{rate}=\frac{1}{t_{1/2}}$$

Avrami parameters calculated from the slopes and interceptions of the Avrami plots in Figure 6 are summarized in Table 5. As seen from the table, the *n* values for neat PLA were 2.46–3.22. PLA/PLA-*g*-MA3/CNF0 and PLA/PLA-*g*-MA0/CNF3 showed *n* values around 2.67–3.21 and 2.59–3.34 that indicates the addition of PLA-*g*-MA or CNF did not change the crystallization mechanism and the crystal geometry growth of PLA. As compared to uncompatibilized PLA/CNF3, the compatibilized PLA/CNF3 nanocomposites exhibited a narrowed range of *n* values between 2.94–3.27 indicates that the addition of PLA-g-MA had a compatibilization effect on the nanocomposites blend [55]. Besides, the *k* value for all the nanocomposite samples increased with the T_c_ and then decreased after reaching a maximum value at 100 °C due to the difficulty of crystal nucleation at an elevated temperature [56].

Other important crystallization kinetics parameters, the crystallization half time (t_1/2_) and crystallization rate (1/t_1/2_), were calculated using Equations (7) and (8). From Table 5, the t_1/2_ for neat PLA were 158.01, 92.72 and 165.59, respectively, when isothermally crystallized at 90, 100 and 110 °C. By solely adding PLA-*g*-MA to PLA blend, the t_1/2_ was reduced to 34.27, 33.22 and 51.33 min. These results show that the functionality of PLA-*g*-MA was not solely as a compatibilizer, but it also assisted in early nucleation. Although PLA-*g*-MA contributes to slow nucleation rate at the beginning, but once the nuclei was nucleated, it promotes rapid crystal growth as can be observed in steep sigmoidal shape of X_t_ vs. t plot of PLA/PLA-*g*-MA3/CNF0 as compared to neat PLA (Figure 5).

**Figure 7:**
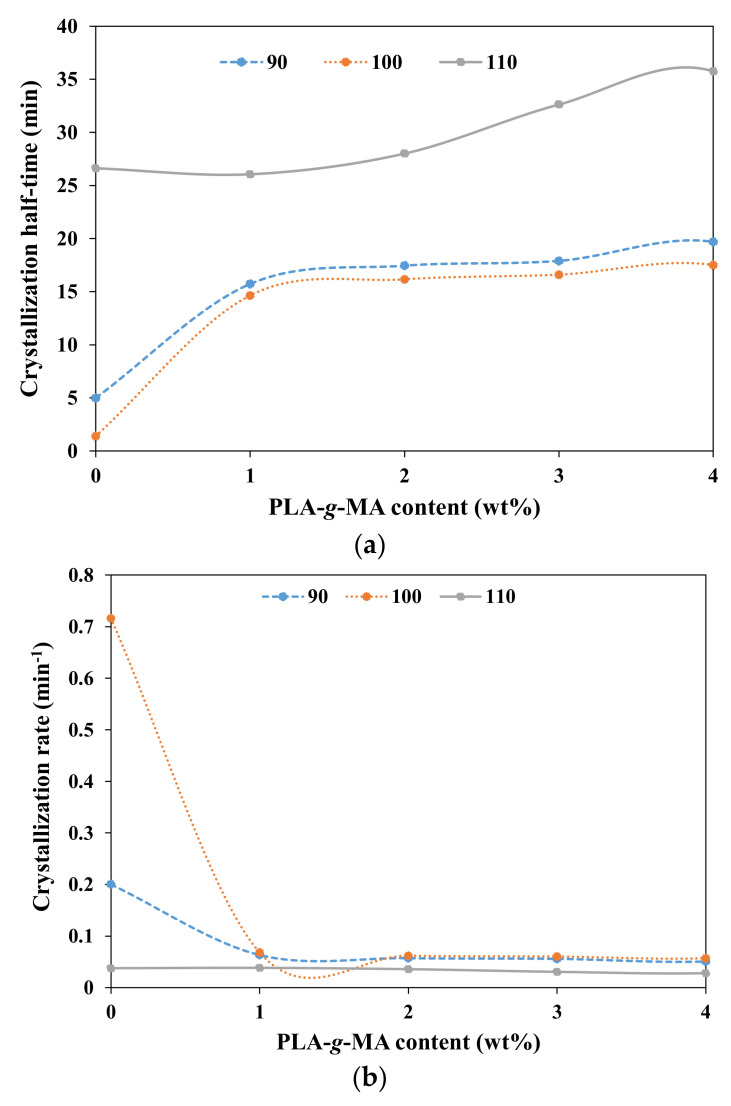
Dependence of (**a**) crystallization half-time and (**b**) crystallization rate on PLA-*g*-MA content for PLA reinforced 3 wt.% CNF melt-crystallized isothermally at 90, 100 and 110 °C.

Both the nucleation and crystal growth processes were however better with the addition of CNF. As shown in Figure 7a,b, the lowest t_1/2_ and the highest crystallization rate are seen when 0% PLA-*g*-MA was used. All PLA/PLA-*g*-MA/CNF3 nanocomposites showed higher crystallization rate as compared to PLA/PLA-*g*-MA. As seen in Table 5 and Figure 7a, the addition of PLA-*g*-MA from 1 to 4 wt.% increases the t_1/2_ values to 19.69, 17.51 and 35.75 min from 4.99, 1.40 and 26.63 min of the PLA/PLA-*g*-MA0/CNF3, respectively, when isothermally melt-crystallized at 90, 100 and 110 °C. These findings indicate that PLA-*g*-MA might be a chain extender between PLA and CNF, resulting in longer polymer chains and higher chain rigidity [57]. As a result, the crystallization half time increased (i.e., 1/t_1/2_ decreased) with the addition of PLA-*g*-MA. Among the T_c_ investigated, more distinct nucleation effects with a minimum t_1/2_ value can be seen at T_c_ = 100 °C for all nanocomposites. This temperature corresponds to the optimum temperature of isothermal crystallization of the nanocomposites similar in PLA/CNF study previously conducted [45]. A similar trend can be seen for crystallization rate as shown in Figure 7b, which shows that the highest crystallization rate was observed for PLA/PLA-*g*-MA0/CNF3 nanocomposites.

Based on the above findings, it can be summarized that despite nucleation effect of PLA-*g*-MA, the effect is not as superior as CNF. Among all, uncompatibilized PLA/CNF3 exhibited the best crystallization rate, suggesting that the presence of PLA-*g*-MA is not necessary for the improvement of crystallization rate of the PLA/CNF nanocomposites.

### 3.4. Mechanical Properties

Mechanical testing was performed to evaluate the nucleation and compatibilization effects of CNF and PLA-*g*-MA on the mechanical properties of PLA/PLA-*g*-MA/CNF nanocomposites. The results for neat PLA, PLA/PLA-*g*-MA3/CNF0 and PLA/PLA-*g*-MA/CNF3 nanocomposites are tabulated in Table 6.

Overall, it is seen that the addition of PLA-*g*-MA increased the Young’s modulus of the PLA, without affecting the tensile strength and elongation at break. Young’s modulus is typically related to the stiffness of the polymer where a more rigid polymer exhibits higher modulus. As seen from Table 6, the Young’s modulus for neat PLA was 2.9 GPa. In comparison to neat PLA, PLA/PLA-*g*-MA3 had much higher Young’s modulus by 280%. As discussed earlier, the addition of PLA-*g*-MA contributed to the restriction of molecular motion when anhydride carboxyl group was grafted onto the PLA. This increases the nanocomposites rigidity, which is being reflected through the high Young’s modulus value [17]. The Young’s modulus recorded was also higher compared to uncompatibilized PLA/CNF3. In the case of PLA/PLA-*g*-MA/CNF3 nanocomposites, it is seen from Table 6 that the Young’s modulus value increased with the PLA-*g*-MA concentration. Elongation at break reduced as opposed to Young’s modulus since rigid material is expected to have lower elongation.

In terms of tensile strength, it is seen that the addition of PLA-*g*-MA slightly reduced the tensile strength. The tensile strength continued to reduce in PLA/PLA-*g*-MA/CNF3 with the increasing amount of PLA-*g*-MA. This could be attributed to the low molecular weight PLA-*g*-MA [18,43]. PLA/CNF3 without PLA-*g*-MA, on the other hand, had a higher tensile strength of PLA/PLA-*g*-MA3. The reinforcement of CNF in PLA matrix without the presence of PLA-*g*-MA significantly increased the strength due to a superior transfer of load from PLA matrix to the CNF, as reported by Abdulkhani et al. [58,59] and Ghasemi et al. [44]. The addition of the PLA-*g*-MA as a compatibilizer is expected to improve the interfacial adhesion between CNF and PLA matrix; however, the tensile strength of the nanocomposites gradually decreased as PLA-*g*-MA content increased.

### 3.5. Morphological Analysis

**Figure 8:**
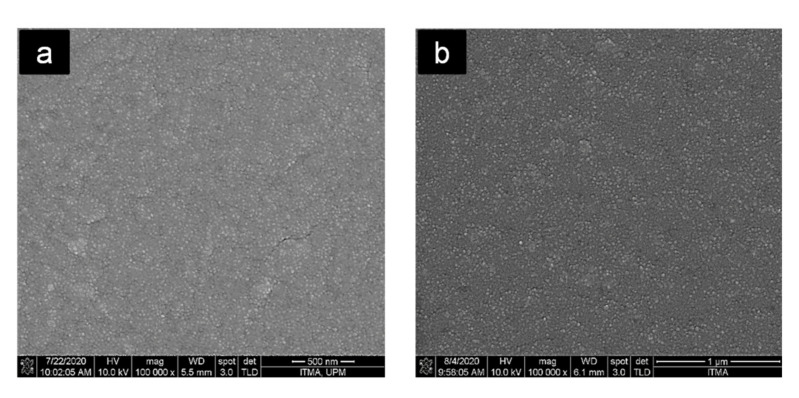

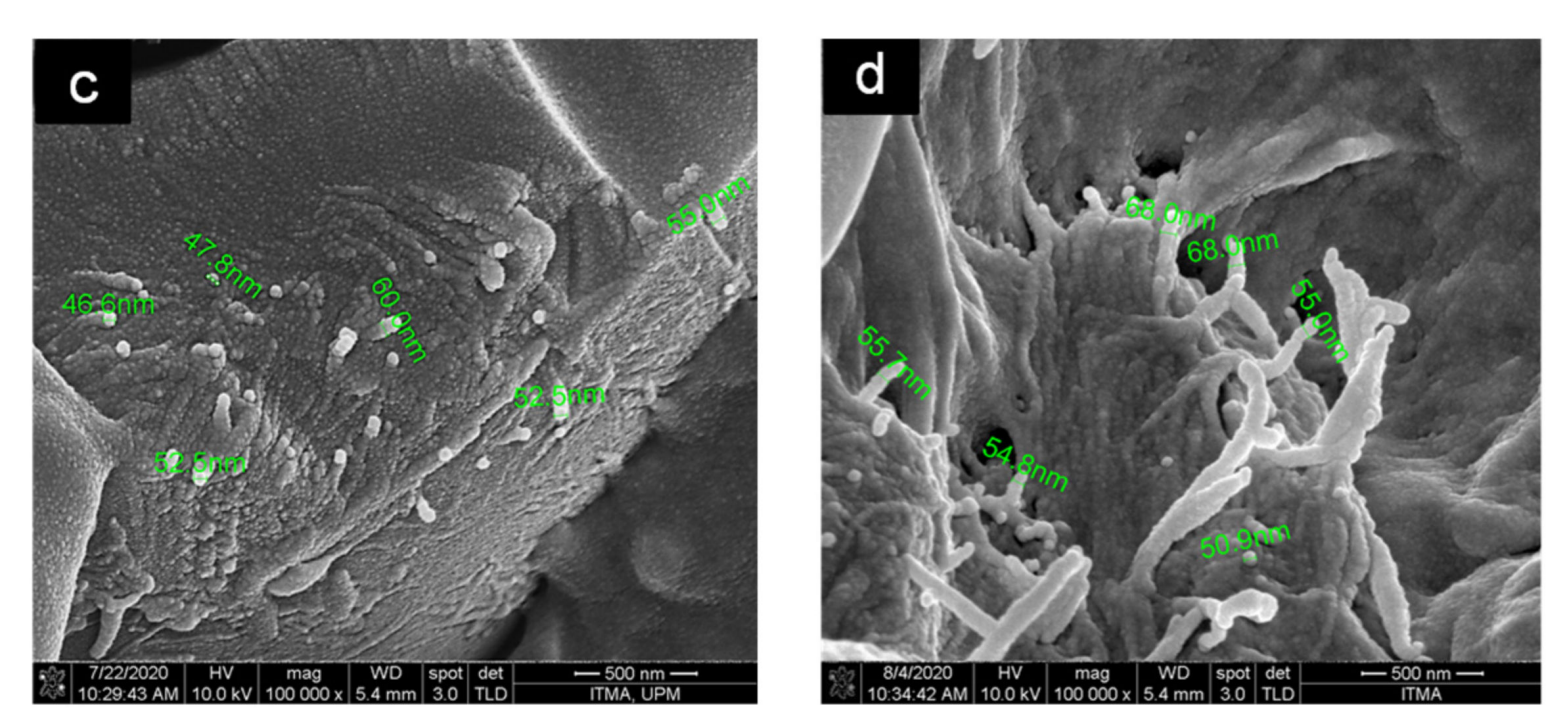
FESEM micrographs of the fractured surfaces of (**a**) neat PLA, (**b**) PLA/PLA-*g*-MA3/CNF0, (**c**) PLA/PLA-*g*-MA0/CNF3 and (**d**) PLA/PLA-*g*-MA3/CNF3 nanocomposites at 100,000× magnification.

The morphologies of the fractured surfaces of the nanocomposites, i.e., neat PLA (Figure 8a), PLA/PLA-*g*-MA3/CNF0 (Figure 8b), PLA/PLA-*g*-MA0/CNF3 (Figure 8c) and PLA/PLA-*g*-MA3/CNF3 (Figure 8d) are shown, respectively.

As seen from Figure 8a,b, a relatively smooth surface can be seen in neat PLA, and PLA/PLA-*g*-MA3/CNF0 indicated the brittle failure characteristics, as the fractures showed little sign of plastic deformation [60]. Moreover, the addition of PLA-*g*-MA did not affect the structure of PLA. For PLA/PLA-*g*-MA0/CNF3, some fiber breakages on the fractured surface could be observed, and no agglomeration can be detected indicates that the CNF was well-blended in PLA matrix even without the presence of PLA-*g*-MA, thus resulted in higher tensile strength than other nanocomposites. For the compatibilized nanocomposites with the same CNF content, the addition of PLA-*g*-MA in the nanocomposites did not seem to affect the samples structure. It was proven that PLA-*g*-MA can also act as a plasticizer in the nanocomposite blends due to the reduction trend of T_g_ and tensile strength as discussed previously in Section 3.2 and Section 3.4.

## 4. Conclusions

The influence of PLA-*g*-MA on nucleating effect of CNF was determined by studying the effect of various PLA-*g*-MA wt.% addition on the crystallization kinetics of PLA/PLA-*g*-MA/CNF3. Data from isothermal crystallization kinetic study at T_c_ = 100 °C showed that the crystallization rate increased from 0.011 to 0.030 min^−1^ as compared to the neat PLA. This shows that PLA-*g*-MA had some effect on the nucleation, even though not as effective as the CNF. When PLA-*g*-MA was added to PLA/CNF3, it was shown that the crystallization rate of the PLA/PLA-*g*-MA/CNF3 decreased as compared to uncompatibilized PLA/CNF3. This shows that the addition of PLA-*g*-MA in PLA/CNF3 was ineffective and unnecessary in improving crystallization kinetics of PLA nanocomposites. Reinforcement of 3 wt.% CNF with the combination of PLA-*g*-MA (1–4 wt.%) in PLA matrix reduced the tensile strength of PLA, but increased the Young’s modulus. Findings from this research revealed that the addition of PLA-*g*-MA did not affect the crystallization property of the PLA/CNF nanocomposites, but only affected the mechanical properties of the nanocomposite samples. Research on the synergistic mechanism of nucleation between CNF and PLA-*g*-MA in the kinetics of biopolymer biodegradation is currently in progress.

## Figures and Tables

**Table 1:** Compositions of PLA, MA and DBPO for PLA-*g*-MA production.

PLA-*g*-MA (MA wt.%)	Composition (wt.%)		
	Polylactic Acid (PLA)	Maleic Anhydride (MA)	Dibenzoyl Peroxide (DBPO)
3	36.25	3	0.75
10	29.25	10	0.75

**Table 2:** Sample codes and compositions of PLA/PLA-*g*-MA/CNF nanocomposites.

Sample	Composition (wt.%)		
	Polylactic Acid (PLA)	Maleated PLA (PLA-*g*-MA)	Cellulose Nanofiber (CNF)
Neat PLA	100	0	0
PLA/PLA-*g*-MA3/CNF0	97	3	0
PLA/PLA-*g*-MA0/CNF3	97	0	3
PLA/PLA-*g*-MA1/CNF3	97	1	3
PLA/PLA-*g*-MA2/CNF3	95	2	3
PLA/PLA-*g*-MA3/CNF3	97	3	3
PLA/PLA-*g*-MA4/CNF3	96	4	3

**Table 3:** Effect of MA content on grafting percentage and efficiency of PLA-*g*-MA.

PLA-*g*-MA (MA wt.%)	MA Grafting (%)	Grafting Efficiency (%)
3	0.15 ± 0.0	3.13 ± 0.4
10	2.10 ± 0.3	13.20 ± 2.2

**Table 4:** Non-isothermal crystallization data of neat PLA and PLA/PLA-*g*-MA/CNF nanocomposites.

Sample	T_g_ (°C)	T_c_ (°C)	T_cc_ (°C)	T_m1_ (°C)	T_m2_ (°C)	∆H_c_ (J/g)	∆H_cc_ (J/g)	∆H_m_ (J/g)	X_c_ (%)
Neat PLA	50.4	106.1	91.3	143.5	152.1	1.3	33.4	35.6	2.3
PLA/PLA-*g*-MA3/CNF0	51.9	96.5	84.5	142.6	152.3	2.0	27.1	33.9	6.7
PLA/PLA-*g*-MA0/CNF3	43.9	114.6	71.0	136.7	147.1	33.6	0.9	42.3	44.2
PLA/PLA-*g*-MA1/CNF3	45.9	112.7	75.5	135.3	146.1	24.5	8.8	35.4	28.4
PLA/PLA-*g*-MA2/CNF3	46.1	115.1	78.5	137.1	147.4	29.0	7.4	35.3	29.8
PLA/PLA-*g*-MA3/CNF3	46.3	113.8	75.8	135.3	146.6	16.8	14.1	41.6	29.4
PLA/PLA-*g*-MA4/CNF3	47.3	113.3	77.9	136.1	147.0	21.1	12.4	41.0	30.5

**Table 5:** Avrami parameters for isothermal crystallization of neat PLA, PLA/PLA-*g*-MA3/CNF0 and PLA/PLA-*g*-MA/CNF3 nanocomposites.

Sample	Tc = 90 °C				Tc = 100 °C				Tc = 110 °C			
	n	k (min^−n^)	t_1/2_ (min)	1/t_1/2_ (min^−1^)	n	k (min^−n^)	t_1/2_ (min)	1/t_1/2_ (min^−1^)	n	k (min^−n^)	t_1/2_ (min)	1/t_1/2_ (min^−1^)
Neat PLA	3.22	5.72 × 10^−8^	158.01	0.006	2.74	2.83 × 10^−6^	92.72	0.011	2.46	2.46 × 10^−6^	165.59	0.006
PLA/PLA-*g*-MA3/CNF0	2.98	1.82 × 10^−5^	34.27	0.029	3.21	9.18 × 10^−6^	33.22	0.030	2.67	1.90× 10^−5^	51.33	0.019
PLA/PLA-*g*-MA0/CNF3	2.59	1.09 × 10^−2^	4.99	0.200	3.11	2.45 × 10^−1^	1.40	0.716	3.34	1.20 × 10^−5^	26.63	0.038
PLA/PLA-*g*-MA1/CNF3	3.04	1.60 × 10^−4^	15.73	0.064	3.12	1.62 × 10^−4^	14.64	0.068	2.95	4.63 × 10^−5^	26.06	0.038
PLA/PLA-*g*-MA2/CNF3	3.10	9.71 × 10^−5^	17.45	0.057	2.98	1.72 × 10^−4^	16.15	0.062	2.99	3.30 × 10^−5^	28.01	0.036
PLA/PLA-*g*-MA3/CNF3	3.27	5.53 × 10^−5^	17.90	0.056	3.10	1.14 × 10^−4^	16.60	0.060	2.99	2.06 × 10^−5^	32.63	0.031
PLA/PLA-*g*-MA4/CNF3	3.21	4.79 × 10^−5^	19.69	0.051	3.27	5.91 × 10^−5^	17.51	0.057	2.94	1.89 × 10^−5^	35.75	0.028

**Table 6:** Mechanical properties of neat PLA, PLA/PLA-*g*-MA3/CNF0 and PLA/PLA-*g*-MA/CNF3 nanocomposites.

Composition	Tensile Strength (MPa)	Young’s Modulus (GPa)	Elongation at Break (%)
Neat PLA	70.8 ± 0.1 ^b^	2.9 ± 0.0 ^f^	2.54 ± 0.1 ^a^
PLA/PLA-*g*-MA3/CNF0	70.0 ± 0.3 ^c^	11.0 ± 0.9 ^d^	2.46 ± 0.4 ^b^
PLA/PLA-*g*-MA0/CNF3	74.1 ± 0.1 ^a^	3.3 ± 0.1 ^e^	2.34 ± 0.1 ^c^
PLA/PLA-*g*-MA1/CNF3	66.1 ± 0.5 ^d^	11.5 ± 0.2 ^c^	2.17 ± 0.3 ^d^
PLA/PLA-*g*-MA2/CNF3	65.9 ± 0.8 ^d,e^	11.6 ± 0.1 ^c^	2.14 ± 0.1 ^d^
PLA/PLA-*g*-MA3/CNF3	65.7 ± 0.1 ^e^	11.7 ± 0.4 ^b,c^	2.11 ± 0.1 ^e^
PLA/PLA-*g*-MA4/CNF3	65.5 ± 0.2 ^e^	11.8 ± 0.1 ^b,c^	2.05 ± 0.3 ^f^

All data are means of 5 replicates ± S.D. The alphabets indicate significant difference (*p* < 0.05) according to Duncan’s Multiple Range Test.
